# Supplementary material for: MetaRibo-Seq measures translation in microbiomes
Source: Nat Commun. 2020 Jun 29;11:3268. doi: 10.1038/s41467-020-17081-z (PMC7324362; doi:10.1038/s41467-020-17081-z)
Supplement: Supplementary file 10 — Supplementary Data 7 [file 41467_2020_17081_MOESM10_ESM.zip › File2/Confidence_High_Taxonomy/305670_out.krona.html]

Javascript must be enabled to view this page.

members
magnitude
magnitudeUnassigned
count
unassigned
taxon
rank

305670\_out

182

2
superkingdom
182

phylum
182
976

200643
class
182

171549
order
182

family
1
1853231

1
genus
283168


SRS024132\_contig\_number\_49914
626933
1
species

181
family
815

181
genus
816

3
species
46506

SRS015960\_contig\_number\_3574SRS020271\_contig\_number\_1868SRS050925\_contig\_number\_2242


SRS018313\_contig\_number\_8995SRS074964\_contig\_number\_878
2
species
1796613


SRS077335\_contig\_number\_1167
1
species
626929


SRS050941\_contig\_number\_contig-100\_588.126470SRS075078\_contig\_number\_6587SRS1055022\_contig\_number\_19347SRS893327\_contig\_number\_4696
818
species
4


SRS064973\_contig\_number\_2748
1
species
2292951

817
1
species

SRS053573\_contig\_number\_76

47678
162
species

SRS011134\_contig\_number\_11517SRS011302\_contig\_number\_15939SRS012273\_contig\_number\_10938SRS012969\_contig\_number\_34583SRS013098\_contig\_number\_40966SRS013521\_contig\_number\_13906SRS013687\_contig\_number\_2802SRS013951\_contig\_number\_39031SRS013965\_contig\_number\_35060SRS014235\_contig\_number\_12039SRS014287\_contig\_number\_20058SRS014313\_contig\_number\_554SRS014412\_contig\_number\_contig-100\_3240.126492SRS014613\_contig\_number\_20989SRS014683\_contig\_number\_16152SRS014923\_contig\_number\_46326SRS014948\_contig\_number\_contig-100\_1134.141428SRS014979\_contig\_number\_contig-100\_1175.180487SRS015663\_contig\_number\_19918SRS015694\_contig\_number\_22568SRS015782\_contig\_number\_34684SRS016018\_contig\_number\_10295SRS016203\_contig\_number\_8315SRS016335\_contig\_number\_contig-100\_6239.172284SRS016989\_contig\_number\_10915SRS017103\_contig\_number\_36063SRS017191\_contig\_number\_16171SRS017433\_contig\_number\_10839SRS017701\_contig\_number\_1747SRS018049\_contig\_number\_994SRS018133\_contig\_number\_10170SRS018427\_contig\_number\_15142SRS018541\_contig\_number\_9803SRS018836\_contig\_number\_21190SRS018936\_contig\_number\_989SRS019030\_contig\_number\_15802SRS019161\_contig\_number\_204SRS019178\_contig\_number\_4914SRS019267\_contig\_number\_886SRS019397\_contig\_number\_3473SRS019601\_contig\_number\_13084SRS019685\_contig\_number\_34898SRS019910\_contig\_number\_16278SRS020233\_contig\_number\_49368SRS020328\_contig\_number\_24809SRS020394\_contig\_number\_998SRS021219\_contig\_number\_8821SRS022071\_contig\_number\_38252SRS023583\_contig\_number\_12986SRS023715\_contig\_number\_3384SRS024492\_contig\_number\_contig-100\_354.74685SRS042966\_contig\_number\_14803SRS043667\_contig\_number\_3592SRS043701\_contig\_number\_10246SRS043768\_contig\_number\_18385SRS043841\_contig\_number\_11289SRS044535\_contig\_number\_6050SRS045004\_contig\_number\_4085SRS045244\_contig\_number\_6907SRS045645\_contig\_number\_3697SRS045826\_contig\_number\_19208SRS046068\_contig\_number\_3473SRS047014\_contig\_number\_23965SRS047044\_contig\_number\_8086SRS047433\_contig\_number\_3202SRS048060\_contig\_number\_contig-100\_1119.82360SRS048164\_contig\_number\_3222SRS049712\_contig\_number\_contig-100\_3059.132833SRS049773\_contig\_number\_3901SRS049995\_contig\_number\_12683SRS050299\_contig\_number\_9911SRS050422\_contig\_number\_27482SRS050998\_contig\_number\_16448SRS051031\_contig\_number\_36570SRS052078\_contig\_number\_877SRS052697\_contig\_number\_38397SRS054905\_contig\_number\_20468SRS055982\_contig\_number\_11502SRS056273\_contig\_number\_26129SRS057480\_contig\_number\_2963SRS058723\_contig\_number\_2111SRS058770\_contig\_number\_16460SRS062427\_contig\_number\_963SRS062654\_contig\_number\_21436SRS063370\_contig\_number\_contig-100\_1926.86051SRS064276\_contig\_number\_28600SRS064557\_contig\_number\_13399SRS064757\_contig\_number\_15658SRS065176\_contig\_number\_6605SRS065397\_contig\_number\_9632SRS065504\_contig\_number\_23847SRS075821\_contig\_number\_22864SRS075878\_contig\_number\_10144SRS077024\_contig\_number\_425SRS077086\_contig\_number\_4129SRS077294\_contig\_number\_11201SRS077392\_contig\_number\_20789SRS077454\_contig\_number\_583SRS077502\_contig\_number\_11734SRS077589\_contig\_number\_contig-100\_1496.54904SRS077849\_contig\_number\_14574SRS078176\_contig\_number\_22684SRS078242\_contig\_number\_contig-100\_1420.193550SRS078419\_contig\_number\_28439SRS078665\_contig\_number\_2538SRS098644\_contig\_number\_45494SRS098827\_contig\_number\_1550SRS100907\_contig\_number\_7456SRS103987\_contig\_number\_16133SRS1041031\_contig\_number\_19769SRS1041039\_contig\_number\_20262SRS1041090\_contig\_number\_2240SRS1041091\_contig\_number\_contig-100\_1601.97191SRS1041092\_contig\_number\_6980SRS1041134\_contig\_number\_14648SRS1041137\_contig\_number\_5626SRS1041138\_contig\_number\_14334SRS1041141\_contig\_number\_14428SRS1041164\_contig\_number\_5577SRS104311\_contig\_number\_11600SRS104400\_contig\_number\_53863SRS104975\_contig\_number\_20821SRS105082\_contig\_number\_12327SRS105153\_contig\_number\_16262SRS1054716\_contig\_number\_11724SRS1055049\_contig\_number\_14062SRS1055076\_contig\_number\_13470SRS142599\_contig\_number\_4689SRS142781\_contig\_number\_8917SRS142890\_contig\_number\_15473SRS143342\_contig\_number\_14910SRS143598\_contig\_number\_2781SRS143722\_contig\_number\_2355SRS143876\_contig\_number\_23155SRS144183\_contig\_number\_37492SRS144362\_contig\_number\_9392SRS144603\_contig\_number\_contig-100\_1769.105403SRS145497\_contig\_number\_117SRS146813\_contig\_number\_23091SRS147346\_contig\_number\_54478SRS147425\_contig\_number\_8128SRS147557\_contig\_number\_31369SRS147977\_contig\_number\_9115SRS148196\_contig\_number\_33009SRS148319\_contig\_number\_11967SRS148424\_contig\_number\_22011SRS148721\_contig\_number\_55058SRS149244\_contig\_number\_6713SRS149784\_contig\_number\_27172SRS149879\_contig\_number\_28058SRS150029\_contig\_number\_9658SRS892882\_contig\_number\_17326SRS893230\_contig\_number\_contig-100\_4971.39773SRS893252\_contig\_number\_15087SRS893279\_contig\_number\_12124SRS893285\_contig\_number\_159SRS893292\_contig\_number\_2001SRS893295\_contig\_number\_12757SRS893300\_contig\_number\_8987SRS893378\_contig\_number\_9857SRS893382\_contig\_number\_contig-100\_2453.38672SRS971276\_contig\_number\_24046

821
species
1

SRS1041118\_contig\_number\_10511

species
1
2292943

SRS015431\_contig\_number\_68817

species
1
246787

SRS053398\_contig\_number\_2882

28116
species
4

SRS015264\_contig\_number\_13520SRS019381\_contig\_number\_16693SRS148091\_contig\_number\_10826SRS149181\_contig\_number\_30271
